# Genomic study of European Clostridioides difficile ribotype 002/sequence type 8

**DOI:** 10.1099/mgen.0.001270

**Published:** 2024-07-25

**Authors:** Ines Dost, Mostafa Abdel-Glil, Søren Persson, Karen Loaiza Conza, Mónica Oleastro, Frederico Alves, Sven Maurischat, Anissa Scholtzek, Christelle Mazuet, Laure Diancourt, Tanel Tenson, Gernot Schmoock, Heinrich Neubauer, Stefan Schwarz, Christian Seyboldt

**Affiliations:** 1 Institute of Bacterial Infections and Zoonoses, Friedrich-Loeffler-Institut, Federal Research Institute for Animal Health, Naumburger Straße 96a, 07743 Jena, Germany; 2 Statens Serum Institut, Dept. Bacteria, Parasites and Fungi, Unit of Foodborne Infections, Artillerivej 5, 2300 Copenhagen, Denmark; 3 National Reference Laboratory of Gastrointestinal Infections, Department of Infectious Diseases, National Institute of Health Doutor Ricardo Jorge (INSA), 1649-016 Lisbon, Portugal; 4 Chief Scientific Office, European Food Safety Authority (EFSA), Parma, Italy; 5 German Federal Institute for Risk Assessment, Department Biological Safety, Max-Dohrn-Str. 8-10, 10589 Berlin, Germany; 6 Institut Pasteur, Université Paris Cité, Centre National de Référence Bactéries anaérobies et Botulisme, F-75015 Paris, France; 7 Institute of Technology, University of Tartu, Nooruse 1, 50411 Tartu, Estonia; 8 Institute of Microbiology and Epizootics, Centre for Infection Medicine, School of Veterinary Medicine, Freie Universität Berlin, 14163 Berlin, Germany; 9 Veterinary Centre for Resistance Research (TZR), Freie Universität Berlin, 14163 Berlin, Germany

**Keywords:** *Clostridioides difficile*, genomes, One Health, pangenome, phylogenetic analysis, RT002, ST8, zoonotic pathogen

## Abstract

*Clostridioidesdifficile* has significant clinical importance as a leading cause of healthcare-associated infections, with symptoms ranging from mild diarrhoea to severe colitis, and possible life-threatening complications. *C. difficile* ribotype (RT) 002, mainly associated with MLST sequence type (ST) 8, is one of the most common RTs found in humans. This study aimed at investigating the genetic characteristics of 537 *C. difficile* genomes of ST8/RT002. To this end, we sequenced 298 *C*. *difficile* strains representing a new European genome collection, with strains from Germany, Denmark, France and Portugal. These sequences were analysed against a global dataset consisting of 1,437 ST8 genomes available through Enterobase. Our results showed close genetic relatedness among the studied ST8 genomes, a diverse array of antimicrobial resistance (AMR) genes and the presence of multiple mobile elements. Notably, the pangenome analysis revealed an open genomic structure. ST8 shows relatively low overall variation. Thus, clonal isolates were found across different One Health sectors (humans, animals, environment and food), time periods, and geographical locations, suggesting the lineage’s stability and a universal environmental source. Importantly, this stability did not hinder the acquisition of AMR genes, emphasizing the adaptability of this bacterium to different selective pressures. Although only 2.4 % (41/1,735) of the studied genomes originated from non-human sources, such as animals, food, or the environment, we identified 9 cross-sectoral core genome multilocus sequence typing (cgMLST) clusters. Our study highlights the importance of ST8 as a prominent lineage of *C. difficile* with critical implications in the context of One Health. In addition, these findings strongly support the need for continued surveillance and investigation of non-human samples to gain a more comprehensive understanding of the epidemiology of *C. difficile*.

## Data Summary

The sequence data for the newly sequenced isolates can be found under the NCBI BioProject accession: PRJNA1091623. The authors confirm all supporting data, code and protocols have been provided within the article or through supplementary data files.

**Impact Statement**

*Clostridioides difficile* is an important enteropathogen that can cause severe infections. *C. difficile* ribotype (RT) 002, mainly associated with MLST sequence type (ST) 8, is one of the most commonly detected RTs in human samples, but is also detected in animals and in the environment. This is the first study that focuses on RT002/ST8 by making use of an exhaustive European isolate collection and all available ST8 genomes from the public database Enterobase. We were able to identify clusters that included strains of human and non-human origin and could prove that RT002/ST8 is a stable but adaptable lineage of *C. difficile*. With this study, we provide a comprehensive genomic overview of this globally prevalent RT lineage and show the importance of a One Health approach to combat human infections with this notorious antimicrobial-resistant pathogen.

## Introduction

The pathogen *Clostridioides* (formerly *Clostridium*) *difficile* causes enteric disease in humans and animals and is responsible for a notable number of fatal infections worldwide [1]. In 2017*,* this antimicrobial-resistant enteropathogen caused 223, 900 infections with hospitalization and 12,800 deaths in the USA alone [2]. In the 2016–2017 Annual Epidemiological Report of the European Centre for Disease Prevention and Control, participating hospitals reported 37,857 *C*. *difficile* infection (CDI) cases, and the annual number of fatal healthcare-associated CDI cases was estimated at 7,864 in the European Union [3]. The Gram-positive, spore-forming, anaerobic bacterium is ubiquitously distributed. It has been detected in the gut of healthy and diseased humans and of different animal species, as well as in different environments and food [1,4]. The species *C. difficile* represents a multiplicity of different PCR ribotypes (RTs) – which are categorized into different types according to polymorphisms in the 16 S–23S rRNA intergenic spacer regions [5]. Some of these lineages have adapted to specific niches, while others are widespread over different habitats [1]. Prominent RT lineages are the hypervirulent RT027, whose successful spread was presumably favoured by its fluoroquinolone resistance [6], and the globally distributed RT078, frequently found in livestock such as pigs and cattle [1,7]. *C. difficile* RT078 is considered to hold potential for zoonotic transmission [8]. Furthermore, there are several emerging RTs showing different epidemiology, with some establishing more locally and others spreading globally. One of these emerging RTs frequently found in humans is RT002 [9], which is typically classified as sequence type (ST) 8 by multilocus sequence typing (MLST) [10,11]. With MLST, the defined segments of the alleles of the seven housekeeping genes that are present in the respective genome — *adk*, *atpA*, *dxr*, *glyA*, *recA*, *sodA* and *tpi* — are determined. RT002 is able to cause severe *C. difficile* infection (CDI) and has been found in humans, different animal species and the environment [1]. It is one of the predominating RTs among *C. difficile* in Asia, especially in Hong Kong [12], and is frequently found in Europe, America and Oceania [13,16], while it has also been reported from Africa [17]. Isolates belonging to RT002 showed an increased sporulation and germination frequency, a higher toxin production capacity for the toxins A and B, an extended stationary phase and a higher heat resistance compared to other RTs [18,20]. Regarding genomic characteristics, RT002 had the lowest intra-ribotype allele difference for clade 1 in a study that compared different typing methods [21]. In a study analysing European *C. difficile* strains via whole-genome sequencing (WGS), there was no within-country clustering reported for RT002, but genetically closely related isolates could be detected over long geographical distances. In addition, one of the investigated RT002 genomes harboured an antimicrobial resistance determinant (the amino acid substitution T82I in GyrA) [22].

To gain a comprehensive overview of the ST8/RT002 lineage, we investigated the genomes of a newly created European collection of *C. difficile* RT002 strains that was complemented with genomes from the public Enterobase database. The Enterobase genomes were derived from different continents and were selected after a dereplication step to include a representative collection in this analysis. We also looked for clusters with relevance in the context of One Health, i.e. clusters containing genomes from human and non-human sources.

## Methods

### ST8/RT002 genome collection

A total of 537 genomes were collected to allow for a comprehensive overview of the genomic characteristics of the RT002 lineage. For that, 298 genomes classified by PCR ribotyping as RT002 were sequenced. The genomes were collected in several European countries within the framework of the FED-AMR project (https://onehealthejp.eu/projects/antimicrobial-resistance/jrp-fed-amr), which includes partners from Statens Serum Institute (SSI) (Copenhagen, Denmark), Federal Institute for Risk Assessment (BfR) (Berlin, Germany), Institute Pasteur (PI) (Paris, France), National Institute of Health Doutor Ricardo Jorge (INSA) (Lisbon, Portugal), University of Tartu (Tartu, Estonia), and Friedrich-Loeffler-Institut (FLI) (Jena, Germany). The *C. difficile* strains were selected from the strain culture collections of the above-mentioned institutes, and were collected between 2010 and 2021. They derived from different sample types (humans, animals, environment and food). Genomic DNA was prepared at the respective institutes and sequenced on Illumina platforms. An overview of all genomes and their respective metadata is provided in Table S1 (available in the online version of this article).

In order to expand the initial set of genomes, we completed our dataset with genome sequences from the public database Enterobase (https://enterobase.warwick.ac.uk/). To this end, all publicly available *C. difficile* genomes from Enterobase were downloaded on 8 May 2022, comprising a total of 24,013 genomes. MLST using the mlst tool (version 2.22.1; https://github.com/tseemann/mlst) and the classical MLST scheme from PubMLST identified 1,437 genomes assigned to ST8 [10]. To reduce redundancies of these genomes due to clonal strains, we then performed a dereplication step at a mash threshold of 0.0005 using Assembly Dereplicator as described elsewhere (version 0.1.0, available at https://github.com/rrwick/Assembly-Dereplicator). Thus, the number of genomes was reduced to 239 unique representative genomes; the final analysed unique representative genomes were selected randomly. The known years of isolation of this genome collection ranged from 2007 to 2022. The indicated sample types belonged to human, environmental and food samples.

In total, 537 genomes from FED-AMR and Enterobase plus one reference genome (*C. difficile* RT002 strain W0003a, accession number NZ_CP025047) were studied. An overview of all genomes and their respective metadata is provided in Table S1. The analysed *C. difficile* ST8/RT002 genomes mainly originated from strains isolated from human samples (88.5 %, 475/537), with 12 (2.2 %) from environmental sources, such as wastewater (*n*=4), soil (*n*=2), manure (*n*=5) and fish (*n*=1) (unclear source classification in Enterobase, therefore categorised as environmental sample), and 11 (2.0 %) from food samples (*n*=10 from chicken meat products and *n*=1 from a potato sample). Six genomes (1.1 %) originated from animal faeces samples [South American camelids (*n*=3), chicken (*n*=1), corn snake (*n*=1) and pig (*n*=1)]. For 33 samples (6.1 %), no metadata information was available.

The majority of the analysed genomes originated from Europe (76.5 %, 411/537), 60 genomes (11.2 %) were from North America, 26 (4.8 %) were from Asia, four (0.7 %) were from Oceania and one (0.2 %) was from South America. No information on the country of origin was available for 35 genomes (6.5 %). The strains corresponding to the 537 genomes were isolated during the time period 2007 to 2021.

### Genome characterisation

All genomes were analysed with a typing pipeline that is under development, available at https://gitlab.com/FLI_Bioinfo/clostyper. This typing pipeline requires the genomes to be analysed in either FASTA or FASTQ format as well as a reference genome of the species. For that, the complete genome assembly of *C. difficile* RT002 strain W0003a (accession number NZ_CP025047) was used as a reference [23]. In a first step, the quality of the raw reads of the FED-AMR genomes was analysed with fastp (version 0.23.2), which reports total counts of sequenced bases and reads, GC content, Phred quality scores, and duplicated sequence regions [24]. The depth of coverage was inferred from the ratio of the total sequenced bases to the size of reference genome (4,075,361 bp). Taxonomic classification with kraken2 (version 2.1.2) [25] followed and raw reads were checked with confindr (version 0.7.4) [26]. Genome assembly was performed with shovill (version 1.1.0) in default mode.

The assembled FED-AMR genomes and genomes retrieved from Enterobase were investigated with SeqKit (version 2.3.1) [27] to report genome statistics values, including total length of genomes, N50 parameter and GC content. Kraken2 (version 2.1.2) was used to confirm taxonomic classification of assembled contigs. The average nucleotide identity (ANI) of the genomes was determined with FastANI (version 1.33) [28] based on comparison to the reference genome W0003a (accession NZ_CP025047).

### Phylogenetic analysis and genome clustering

To reveal phylogenetic relationships, snippy (version 4.6.0) was used to perform a core genome single-nucleotide polymorphism (cgSNP) analysis based on the reference strain (accession NZ_CP025047, W0003a). Prophages and large repetitive regions were then identified in the reference genome with Phaster (http://phaster.ca/) and nucmer (version 3.1; parameters: -L 1000 -I 90) [29,30]. SNP positions located in these regions were masked. An alignment of the core genome SNPs was then generated with snippy-core. The hard definition of a core SNP was applied, allowing for no gaps. Subsequently, recombination sites were filtered out from the alignment with Gubbins (version 3.2.1) [31]. The resultant recombination purged alignment was used for phylogenetic and clustering analyses. For that, RAxML-NG (version 1.1) [32] was used to build a phylogeny and the final phylogenetic tree was midpoint rooted with Treebender (version 1.1.0) [33].

For genome clustering, the pairwise SNP distances of the core genome were calculated from the recombination purged alignment. The hclust function from the R package stats (version 3.6.3) was used to perform hierarchical clustering of the pairwise SNP distances using thresholds (0, 2, 5, 10, 20, 50, 100, 200 and 1,000 SNPs). This clustering approach was then augmented using fastBAPS version 1.0.8, which performs hierarchical BAPS clusters based on sequence alignment [34].

Next to the cgSNP analysis, a core genome MLST (cgMLST) analysis was performed with chewBBACA version 3 [35] using the cgMLST scheme according to Bletz *et al*. [36] (https://www.cgmlst.org/ncs/schema/12556067/). The pairwise cgMLST allelic distances were calculated and hierarchical clusters based on these distances were detected (thresholds of 0, 3, 6, 10, 20, 50, 100, 200, 1,000 different alleles). Phylogenetic trees were visualized with iTOL (v. 6.6) [37].

### Pangenome analysis

Core and total genes were counted with the software panaroo (version 1.3.0) using default settings in strict mode [38]. For that, all FASTA data were annotated with Bakta (version 1.5.1) [39] and the gff3 files were used to feed the panaroo software. Analysis of the pangenome was performed with PanGP (version 1.0.1) [40] as described elsewhere [41].

### Genetic determinants of antimicrobial resistance and virulence

To search for antimicrobial resistance (AMR) genes, the AMRFinderplus (version 3.11.2, database version 2022-12-19.1) [42] and ABRicate (version 1.0.1) tools [with the databases CARD, NCBI, Resfinder, Megares and ARG-ANNOT (last update on 10 January 2023)] were used. Furthermore, a deeper analysis of the AMR genes and their genetic environment was performed with the software Geneious Prime 2021.0.1. In addition, ABRicate software was also used for the detection of virulence genes with the virulence factor database (VFDB) [43]. For the detection of the CDT genes, the following accession numbers were used: AAF81760 (CDTa) and AAF81761 (CDTb).

### Identification of mobile genetic elements

In a last step, mobile genetic elements (MGEs) including transposons, plasmids and prophages were determined. For that, a customized database was created containing these MGEs described in the literature for *C. difficile*. The accession numbers used to build this customized database are available in Table S2. To search for these MGEs, we used the tool SRST2 v0.2.0 to identify these elements directly from the raw sequencing reads [44]. A coverage cutoff value of 70 % was used for the transposons and 50 % for the plasmids and phages.

### Cross-sectoral clusters of the ST8/RT002 lineage

With the help of the cgSNP results, we searched for clusters of the ST8/RT002 lineage that encompass both human and non-human sources (referred to later as zoonotic clusters) that had a maximum threshold of two SNPs. The threshold of two SNPs in the core genome was used as the molecular clock of *C. difficile* estimated to count 1.4 mutations per year [45].

To obtain a full overview for all epidemiologically related clusters, we additionally performed a cgMLST analysis with all FED-AMR RT002 (*n*=298) and Enterobase ST8 genomes (*n*=1,437). Here, all genomes of the Enterobase database that were assigned to ST8 were included. A threshold of three for the allelic difference was used [21]. Sample type and origin of the genomes were determined, and cross-species clusters that contained at least one non-human and one human isolate were searched for. In the next step, antimicrobial resistance determinants in these genomes were investigated with AMRFinderplus and ABRicate as described above.

## Results

### Concordance between PCR ribotyping and MLST in RT002 strains of *C. difficile*

Among the 298 genomes characterized as RT002 using PCR-based ribotyping, 296 were assigned to the MLST ST8 representing 99.3 % of the investigated genomes. Only two RT002 genomes were assigned to ST309 and ST611 due to allele variations in *glyA* (allele 49 instead of allele 6) and *recA* (allele 47 instead of allele 1), respectively. These results indicate a strong correlation with a remarkably high level of agreement between PCR ribotyping and MLST for RT002 strains of *C. difficile*.

### Global phylogeny of ST8/RT002 genomes

To explore the phylogenetic structure of the ST8/RT002 lineage, we performed a cgMLST and a cgSNP analysis of all 538 genomes (298 genomes collected in the course of the FED-AMR project, 239 dereplicated non-clonal genomes from the public database Enterobase and one reference).

With the cgMLST analysis, the analysed ST8/RT002 genomes revealed a median allelic difference of 35 alleles and a mean value of 44 differing alleles. The highest allelic difference was 503 between two genomes of this sequence type. Eleven clusters with zero allelic differences were detected (nine clusters with two genomes, two clusters with three genomes). Nearly all genomes could be assigned to one main branch by applying a threshold of 200 for the allelic distance. Only four genomes, three originating from Asia and one from Oceania, had to be assigned to a separate branch. With a threshold of 50 differing alleles, one main cluster was detected that contained 97.4 % (523/537) of all analysed genomes, including genomes from all investigated continents. Even with a threshold of 20 differing alleles, 74.3 % (399/537) of the investigated genomes had to be assigned to one cluster. With a threshold of three differing alleles that is recommended to identify epidemiologically related genomes [21], 29 cgMLST clusters of at least two genomes each could be detected. The largest cluster contained 19 genomes.

**Fig. 1.**
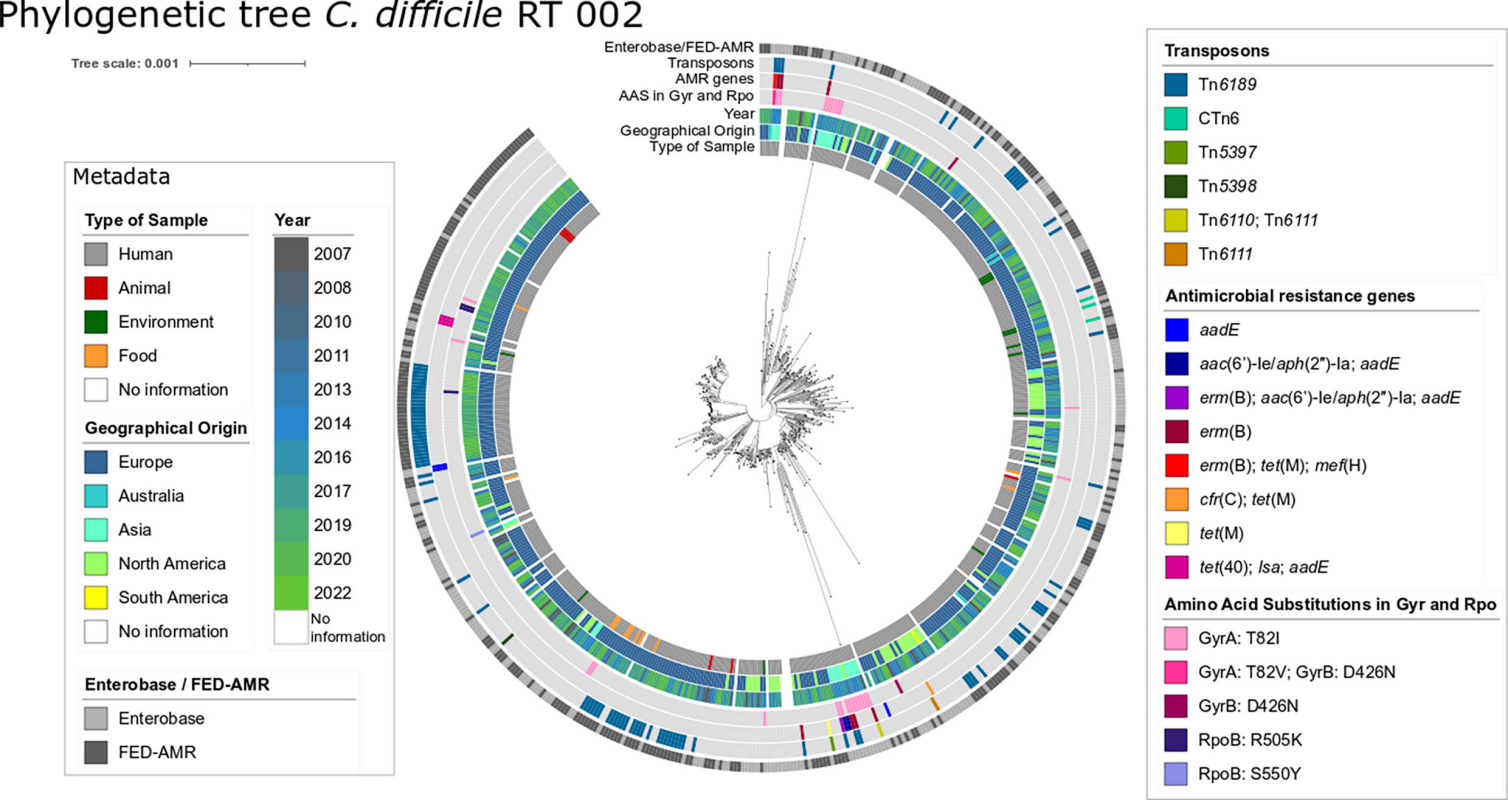
Phylogenetic tree (cgSNP) of *C. difficile* ST8/RT002. In this tree, 298 FED-AMR genomes, 239 dereplicated (see Methods section, ‘ST8/RT002 genome collection’) Enterobase genomes and one reference genome (W0003a, accession number NZ_CP025047) were included. There is no obvious clustering of antimicrobial resistance determinants or transposons in special branches of the tree, and neither is there clustering according to the geographical origin or the sample type to special branches. It is striking that 73 % (19/26) of Asian genomes harbour antimicrobial resistance determinants. The phylogenetic tree was created with iTOL (v. 6.6) [37]. A web version of the tree can be accessed at https://itol.embl.de/tree/193221152140881717331704.

With the cgSNP analysis, a pairwise SNP distance up to 316 SNPs was detected with an average distance of 71 SNPs in the core genome. The median cgSNP distance was 67 SNPs between any two genomes. With a cgSNP threshold of 100 SNPs, nearly all genomes were assigned to the same cluster (99.4 %; 534/537). The three divergent genomes originated from Asia and were assigned to a single separate cluster each. With a threshold of 50 SNPs, 19 clusters were detected. The main cluster harboured 87.9 % (472/537) of the genomes. By applying a threshold of 10 SNPs, which was used as a threshold for genetically distinct isolates [46], 259 singletons and 55 clusters with two or more genomes could be detected. To detect clonal clusters, a threshold of two SNPs was used [45]. A total of 411 singletons and 44 clusters containing two or more genomes were detected. The biggest cluster contained 19 genomes (Fig. 1).

### Pangenome analysis

**Fig. 2.**
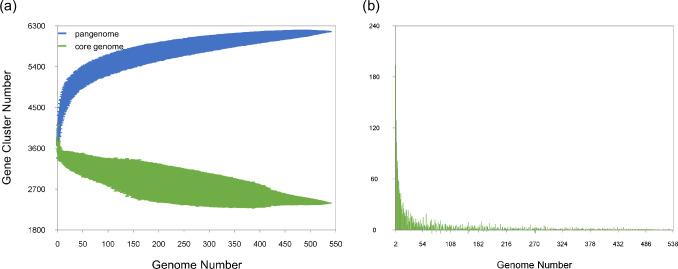
Pangenome and core genome of *C. difficile* ST8/RT002. The pangenome analysis was conducted with the software panaroo. (a) The total number of genes of the pangenome (blue) and the core genome (green) of *C. difficile* ST8/RT002 were calculated. The pangenome of 538 investigated genomes comprised 6,173 genes. (b) The number of new gene clusters per sequentially added genome are depicted as green columns.

A total of 6,173 genes were predicted to comprise the pangenome of *C. difficile* ST8/RT002. The core genome (genes present in more than 95 % of the genomes) represented 55.7 % (3,438/6,173) of the pangenome, while the accessory genome comprised 44.3 % (2,735/6,173). The pangenome was predicted to be in an open state as the number of gene clusters increased with added genomes (Fig. 2). This is also evidenced by the curve analysed using a power law regression model [47,48] showing that the fitting parameter Bpan value was 0.05, indicating an open structure of the pangenome [49].

Functional annotation of the pangenome showed that 62.37 % (3,850/6,173) of the sequences were categorised into functional cluster of orthologous groups (COG) categories. Specifically, 82.11 % (2,823/3,438) of the core genome sequences and 37.55 % (1,027/2,735) of the accessory genome sequences were assigned to these categories. Interestingly, the COG category X, which includes mobilome elements, such as prophages and transposons, was represented by 176 genes in the pangenome. This category was significantly overrepresented in the accessory genome including 165 genes, i.e. 93 % of the total genes in this category, compared to only 11 genes in the core genome. Conversely, the functional clusters of orthologous groups of metabolisms were predominantly present in the core genome and accounted for 92.9 % of this category, in contrast to only 7.07 % in the accessory genome. This emphasises a significant enrichment of metabolic functions in the core genome compared to the accessory genome (Table S3).

### Virulence factors

A total of 13 virulence factor genes were detected, according to the used database. In all genomes, CD0873, CD2831, and *fbpA*/*fbp68* (all coding for adherence factors), *groEL* (molecular chaperon and involved in cell adherence), and the genes *zmp1* and *cwp84* (coding for a zinc metalloprotease and a cell wall-binding cysteine protease, respectively) were detected [50,55]. The genes CD3246 and *cbpA*, coding for a surface protein that is involved in biofilm formation and an adhesin [56,57], respectively, were detected in 99.8 % (536/537) of the genomes. Parts of *cwpV* (coverage 30.6–32.1 %), which codes for a haemagglutinin/adhesin, were detected in 98.9 % (531/537) of the genomes. The toxin gene *tcdB* was detected in all genomes (coverage range 30.63–100 %) and *tcdA* (coverage range 32.05–100 %) was found in 99.8 % (536/537) of the genomes. Incomplete binary toxin genes *cdtA* and *cdtB* were detected in 99.4 % (534/537) and 100 % (537/537) of the genomes, respectively. As RT002 is known to not produce binary toxin, further analysis of the detected binary toxin gene elements was performed. It could be shown that the parts of the genes were absent where PCR primers hybridize [58]. Coverage values of the detected virulence factors for all genomes can be found in Table S4.

### Antimicrobial resistance determinants

#### Common antimicrobial resistance determinants in *C. difficile* ST8/RT002

Several antimicrobial resistance genes were detected in almost all analysed genomes. An overview of the results obtained for different databases can be found in Tables S5−S10. In all 537 genomes, the multidrug efflux transporter *cdeA* was present, which confers norfloxacin and ciprofloxacin resistance in *Escherichia coli* [59]. In addition, the beta-lactam sensor/signal transducer *blaR1* and chromosomal class D beta-lactamase genes were detected in all genomes. These are known to confer intrinsic beta-lactam resistance in *C. difficile* [59]. Mostly *bla*_CDD-1_ was found (99.6 %; 535/537) and *bla*_CDD-2_ was present in three genomes. There was one genome that harboured both beta-lactamase genes. A *van* gene cluster, composed of the genes for the d-Ala-d-Ser ligase *vanG*, the transcription regulator *vanRC*, the sensor kinase *vanSC* and the serine racemase *vanTC*, was present in all genomes [60]. The gene *vanZA*, part of the *vanA* operon that confers teicoplanin resistance [60], was detected in 91.4 % (491/537) of the genomes. A gene related to the *vga*(D) gene family [detected by AMRfinderplus with hidden Markov models (HMMs)], coding for an ABC-F-type ribosome protection protein, was found in 99.1 % (532/537) of the genomes. The gene *vga*(D) confers resistance to streptogramin A antibiotics in *Enterococcus faecium* [61].

### Macrolide, lincosamide and streptrogramin resistance

Next to the *vga*(D) gene, the *erm*(B) gene, coding for resistance to macrolides, lincosamides and streptogramin B antibiotics, was present in nine genomes, all from Asia, except one from Europe. Further analysis revealed that it was mostly located on transposon Tn*6189* (8/9). The putative MGE that harboured *erm*(B) in CLO_EA0075AA (from Asia) could not be assigned to a transposon in our database. Comparative analysis revealed that it was composed of elements of Tn*6189* and Tn*6218* (Fig. S1).

The gene *cfr*(C) gene was detected in one genome, CLO_AA1673AA, from North America. The putative MGE was built in a similar way to Tn*6189*. After aligning the putative MGE to T*n6189*, it was shown that the putative element consists of three parts with identity values of >90 % of the outer parts. The third part in the middle of the element, which contained *cfr*(C), showed a low identity of 19.4 % (Fig. S2).

The gene *mef*(H) coding for the macrolide efflux protein was detected in CLO_DA9723AA, together with a *tet*(M) gene (described below).

In three genomes, an *lsa* gene (coding for an ABC-F ribosome protection protein) was detected. The closest reference sequence was *lsa*(B) (WP_011133547.1; the detected genes showed 68.29 % identity to this reference sequence). It was detected on a putative MGE together with *tet*(40) and *aadE*.

### Tetracycline resistance

The tetracycline resistance gene *tet*(M) was found in three genomes (CLO_AA1673AA, from North America, CLO_DA9723AA, from Asia, and ST8-SSI-h191-19, from Europe) on (putative) MGEs. In ST8-SSI-h191-19, *tet*(M) was located on Tn*5397* (with deletion of the group II intron; Fig. S3). The putative MGEs of CLO_AA1673AA and CLO_DA9723AA showed a similar composition (Fig. S4). The MGE of CLO_DA9723AA was revealed to be a Tn*6944* transposon (GenBank accession number BK013348.1) and next to *tet*(M) harboured the macrolide efflux gene *mef*(H) [62], while the MGE of CLO_AA1673AA was identified as Tn*6944*-like element (88.4 % identity). In three genomes (ST8-SSI-h048-18, ST8-SSI-h188-19 and CLO_BA4294AA, all from Europe), a putative MGE was found containing a resistance operon comprising the genes *tet*(40), an *lsa* gene related to *lsa*(B) and *aadE*.

### Aminoglycoside resistance

Apart from the three genomes that contained *aadE* together with *tet*(40) and *lsa*, similar MGEs containing *aadE* (CLO_DA7520AA and ST8-SSI-h020-18, both from Europe, and DSM29687, no information about the origin) or *aadE* together with *aac*(6′)-Ie/*aph*(2″)-Ia (CLO_DA9523AA, CLO_DA9620AA and CLO_DA9627AA, all from Asia) were detected in six genomes (Fig. S5).

### Fluoroquinolone resistance

**Fig. 3.**
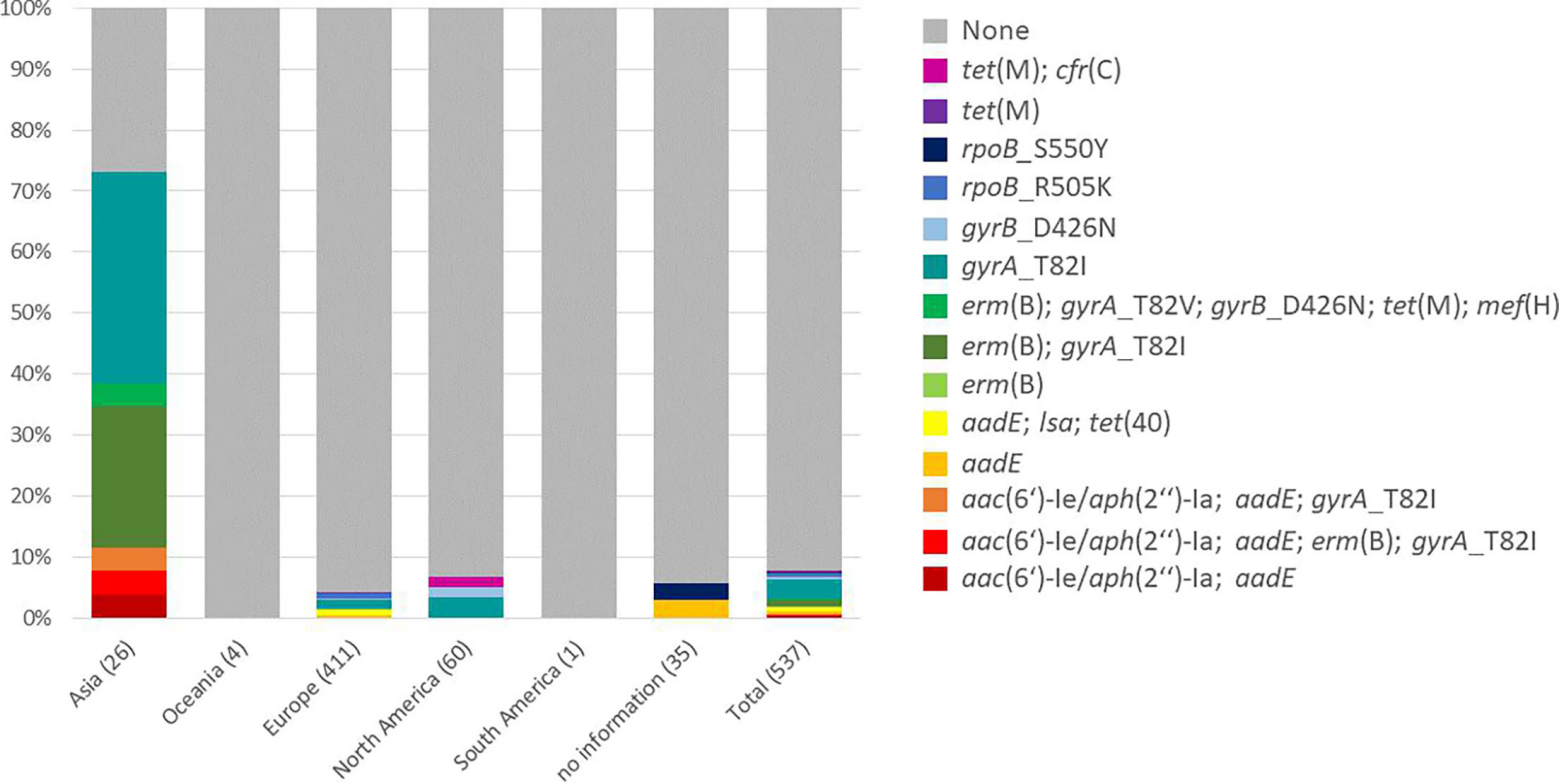
Antimicrobial resistance profiles of selected resistance determinants in ST8/RT002 per continent. All detected antimicrobial resistance (AMR) determinants are included except for those commonly found in ST8/RT002 [*cdeA*, *blaR1*, *bla*_CDD-1_, *bla*_CDD-2_, *van* gene cluster (*vanG*, *vanRC*, *vanSC* and *vanTC*), *vanZA* and *abc-f* (*vga*(D)-related)]. The number of investigated genomes can be found in parentheses after the respective continent. AMR determinants were detected in 73.1 % (19/26) of the Asian ST8/RT002 genomes, conferring resistance to fluoroquinolones, macrolides, tetracycline and aminoglycosides, while 4.1 % (17/411) of the European and 6.7 % (4/60) of the North American genomes harboured AMR determinants.

The amino acid substitution T82I in the quinolone resistance determining region (QRDR) of GyrA was detected in 26 strains. One genome (CLO_DA9723AA) harboured both the substitutions T82V in GyrA and D426N in GyrB, while two genomes (CLO_CA0427AA and ST8-SSI-h031-21) harbour only the substitution D426N in GyrB. It was striking that 18 of the 26 isolates (69 %) harbouring GyrA amino acid substitutions originated from Asia (Fig. 3).

### Rifampicin resistance

Two different amino acid substitutions were found in RpoB. R505K was detected in three isolates from Europe (more specifically from Denmark) (ST8-SSI-h039-18, ST8-SSI-h040-18 and ST8-SSI-h217-21), and S550Y was detected in one isolate (CLO_AA1422AA, no information about the origin).

### Antimicrobial resistance in non-human *C. difficile* RT002 isolates

Besides the AMR determinants that were found in most of the genomes [*cdeA*, *van* gene cluster, *vanZA*, beta-lactamases and the *vga*(D)-related gene], there was only one *C. difficile* genome (CLO_EA0400AA) obtained from a non-human sample that harboured an AMR determinant (amino acid substitution T82I in GyrA), corresponding to a wastewater sample from the UK.

### Mobile genetic elements

The transposon Tn*6189* could be detected in 100 genomes, but only in eight cases was the *erm*(B) gene present. Tn*6189*-like elements lacking *erm*(B) were only found in FED-AMR isolates, mostly from Denmark (82/92). The first genome harbouring the *erm*(B)-lacking Tn*6189*-like element was isolated in Denmark in 2010. In the next years, only single or few genomes with this transposon were detected in this country (2010 *n*=1, 2012 *n*=1, 2015 *n*=1, 2016 *n*=4); from 2018 to 2021, the number of isolates with this MGE increased to 15 - 23 per year (2018 *n*=15, 2019 *n*=22, 2020 *n*=15, 2021 *n*=23). In France, Germany and Portugal, only few genomes that contain this MGE were detected, the first one in Portugal in 2014. CTn*6*-like elements were present in three genomes, 22S0376, IP-e-D19MD028 and ST8-SSI-h216-21. In ST8-SSI-h191-19, a Tn*5397*-like element was detected without the group II intron. A Tn*5398*-like element without the two copies of *erm*(B) was found in CLO_CA0315AA. In CLO_EA0075AA, Tn*6110*- and Tn*6111*-like elements were detected. Another Tn*6111*-like element was present in CLO_FA4322AA.

**Fig. 4.**
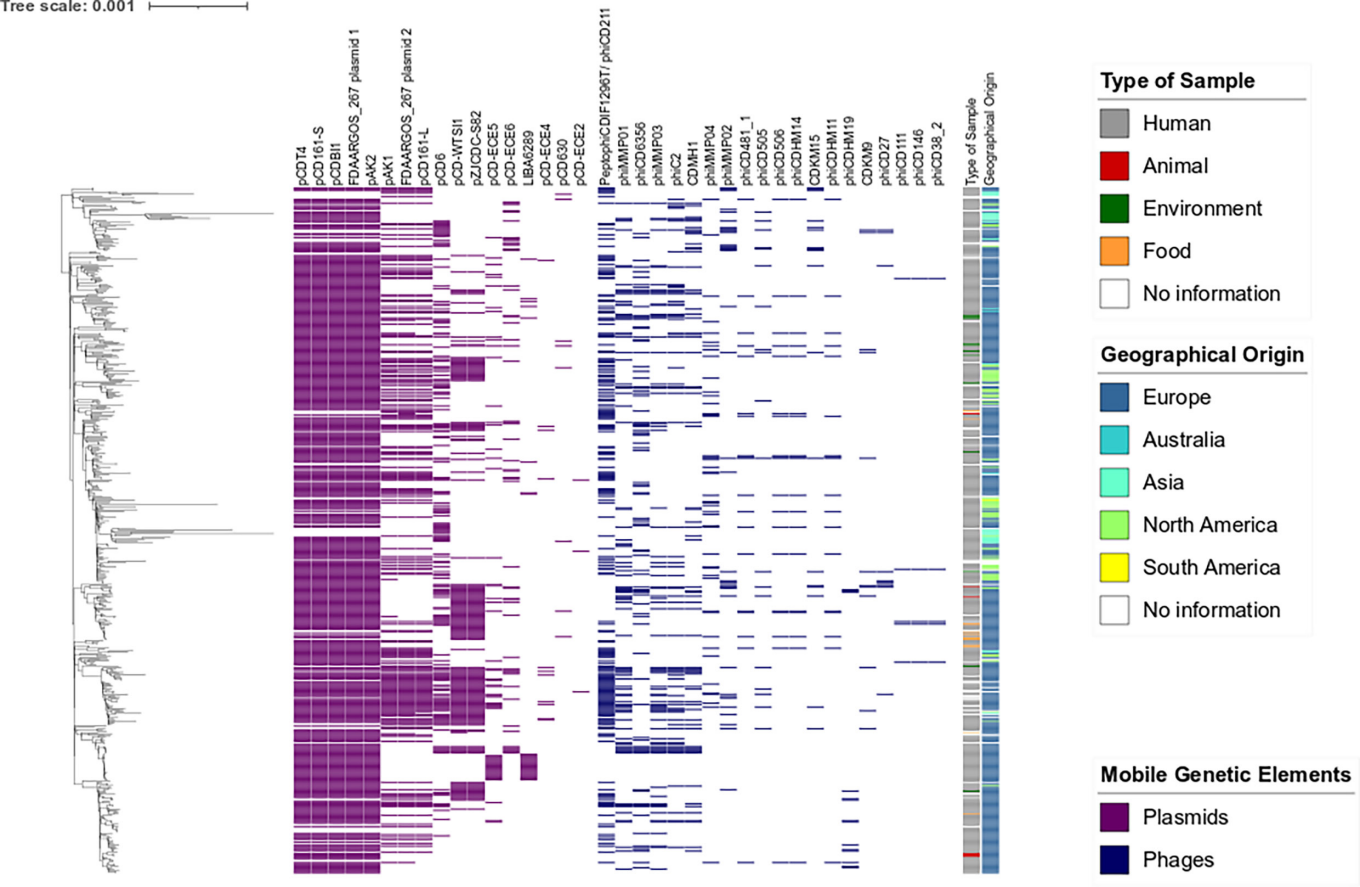
Phylogenetic tree of ST8/RT002 (cgSNP) with detected plasmids and prophages. In this tree, 298 FED-AMR genomes, 239 dereplicated (see Methods section, ‘ST8/RT002 genome collection’) Enterobase genomes and one reference genome were included. This phylogenetic tree was created according to results of the cgSNP analysis. It shows the occurrence of plasmids (purple) and prophages (blue) for each genome. The phylogenetic tree was created with iTOL (v. 6.6) [37].

Different plasmids from our database could be detected. The plasmid content did not differ in genomes from different continents. In addition, the investigated FED-AMR genomes showed a similar profile to the genomes from Enterobase, even though the two genome collections were created with different approaches (see the Methods section for further information) (Table 1). The most common plasmids were pCDBI1 (45.3 kb), pAK2 (56.4 kb), pCDT4 (48.6 kb), pCD161-S (48.6 kb) and plasmid 1 of *C. difficile* strain FDAARGOS_267 (45.2 kb). They were detected in 471 genomes (87.7 %, 471/537). These plasmids share >96 % sequence identity, possibly indicating one plasmid family (Fig. S6b). In 208 genomes, at least one of these plasmids was detected, and in 95.2 % of these (198/208) all three plasmids were present. No obvious clustering of any plasmid in special branches of the phylogenetic tree (cgSNP) could be detected (Fig. 4).

**Table 1.** Abundance of plasmids detected in RT002/ST8 genomes given in percentage and absolute numbers per continent

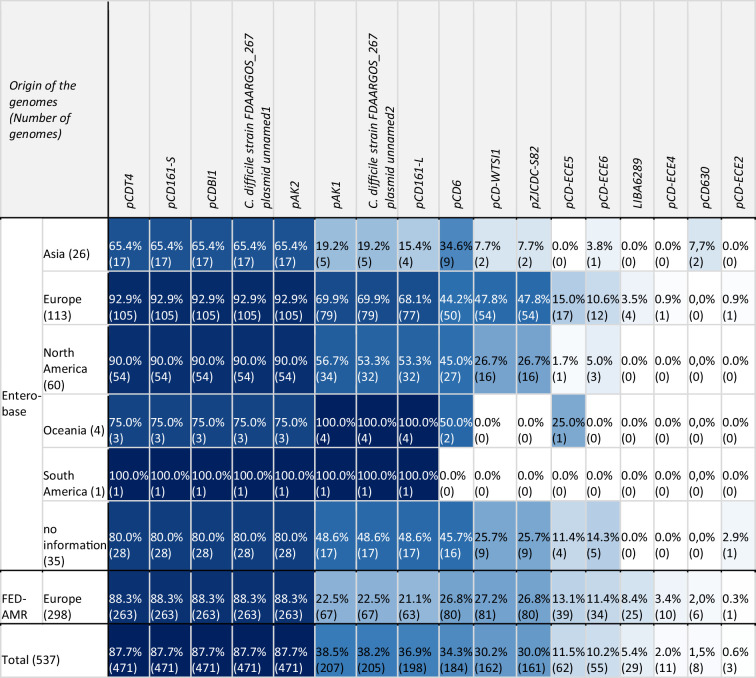 ­

The results in this table are coloured in blue tones according to the abundance of plasmids detected in genomes per continent (dark blue, 100 %; white, 0 %).

A total of 20 prophages or parts of them from the customized database were detected. The abundance of prophages detected in the investigated genomes is shown in Table 2. The prophage profiles did not differ in genomes from different continents. Moreover, the investigated FED-AMR genomes showed a similar profile to the genomes from Enterobase, even though the two genome collections were created with different approaches (see the Methods section for further information). With 38.2 %, PeptophiCDIF1296T (also named phiCD211) was the most present prophage. Similar to plasmids, the average nucleotide identities were calculated for the prophages of the customized database. A high concordance was detected for phiCDHM11, phiCDHM13 and phiCDHM14 (>99.9 %) (Fig. S6c). In all phiCDHM14-positive genomes (*n*=19), either phiCDHM11 (18/19) or phiCDHM13 (1/19) was present. The prophage phiCDHM19 was only found in two cgSNP clusters (threshold 20 SNPs, cluster 121_FED-AMR and 116_FED-AMR). The respective genomes originated from samples from Denmark (*n*=13, human) and France (*n*=1, manure). It was the only prophage for which a clustering in special branches of the phylogenetic tree could be detected. These cgSNP clusters also contained genomes without detected phiCDHM19 (Fig. 4).

**Table 2.** Abundance of prophages detected in RT002/ST8 genomes given in percentage and absolute numbers per continent

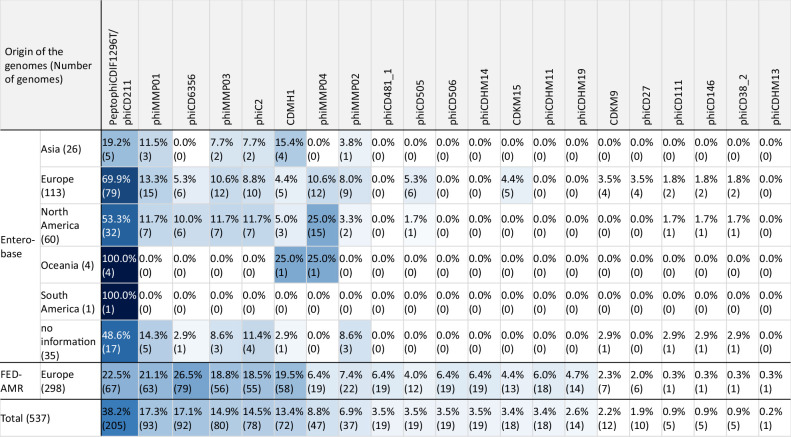 ­

The results in this table are coloured in blue tones according to the abundance of prophages detected in genomes per continent (dark blue, 100 %; white, 0 %).

### Cross-sectoral clusters of the ST8/RT002 lineage

Within the cgSNP analysis considering a threshold of two SNPs, one cross-sectoral cluster could be detected (cgSNP cluster 325) (Table 3). This cluster contained eight genomes from human cases in Denmark (*n*=2) and France (*n*=1) and chicken meat product samples collected in Germany (*n*=4). No information was available for the Enterobase genome. Four of these genomes were even identical according to cgSNP [0 SNPs difference, cluster 339_TH0 (threshold 0), chicken and human samples]. Even though they form a clonal cluster [45], they showed different profiles of the investigated MGEs, except from a French human sample and a German chicken sample showing an identical MGEs profile. No antimicrobial resistance determinants were detected, except for *cdeA*, *van* gene cluster, *blaR1* and *bla*_CDD-1_, *vanZA* and *the vga*(D)-related gene.

**Table 3.** Cluster with One Health importance: cgSNP cluster 325

Cluster no.		ID	Sample	Origin and year	Tn	Plasmids	Prophages
TH2	TH0						
325	335	RT002-BfR-f2	Chicken wings	DE, 2018	Tn*6189*	pZJCDC-S82; pCD-WTSI1	–
	336	RT002-BfR-f3	Chicken drumstick	DE, 2018	Tn*6189*	pCD630; pCDT4; pCD161-S; pCDBI1; pZJCDC-S82; pCD-WTSI1; strain FDAARGOS_267 plasmid unnamed 1; pAK2	–
	337	IP-h-2017–220	Human	FR, 2017	Tn*6189*	pCD630; pCDT4; pCD161-S; pCDBI1; pZJCDC-S82; pCD-WTSI1; strain FDAARGOS_267 plasmid unnamed 1; pAK2	–
	338	CLO_EA8355AA	Unknown	DE, Unknown	–	pCD161-L; pZJCDC-S82; pCD-WTSI1; strain FDAARGOS_267 plasmid unnamed 2; pAK1	PeptophiCDIF1296T/ phiCD211
	339	ST8-SSI-h259-15	Human	DK, 2015	Tn*6189*	pCD630; pCDT4; pCD161-S; pCDBI1; pZJCDC-S82; pCD-WTSI1; strain FDAARGOS_267 plasmid unnamed 1; pAK2	phiCD481_1; phiCD506; phiCDHM11; phiCDHM14; phiMMP04
		ST8-SSI-h252-16	Human	DK, 2016	Tn*6189*	pCDT4; pCD161-S; pCDBI1; pZJCDC-S82; pCD-WTSI1; strain FDAARGOS_267 plasmid unnamed 1; pAK2	CDMH1; phiC2; phiMMP01; phiMMP03
		RT002-BfR-f5	Chicken wings	DE, 2018	Tn*6189*	pZJCDC-S82; pCD-WTSI1	–
		RT002-BfR-f9	Chicken wings	DE, 2018	–	pCD161-L; pZJCDC-S82; pCD-WTSI1; strain FDAARGOS_267 plasmid unnamed 2; pAK1	PeptophiCDIF1296T/ phiCD211

TH2:, cgSNP cluster with a threshold of two SNPs; TH0:, cgSNP cluster with a threshold of zero SNPs.

To obtain a full overview of all epidemiologically related clusters, we performed a cgMLST analysis with all FED-AMR RT002 (*n*=298) and all Enterobase ST8 genomes (*n*=1,437). The analysed Enterobase ST8 genomes represented 6 % (1,437/24, 013) of the Enterobase *C. difficile* collection at the time of analysis. The origin and year of isolation of the ST8 genomes are described in Fig. S7.

The vast majority of Enterobase ST8 genomes originated from human samples (85 %; 1,217/1,437). Only 41 (3 %) of ST8 genomes were from non-human samples, including dog (*n*=2), mouse (*n*=1), swine (*n*=9), chicken manure (*n*=3), fish (*n*=1), soil/dust (*n*=1), wastewater (*n*=22) and potato (*n*=2) samples. For 16 genomes, ‘laboratory’ was given as source and for 163 genomes, no information was available.

**Fig. 5.**
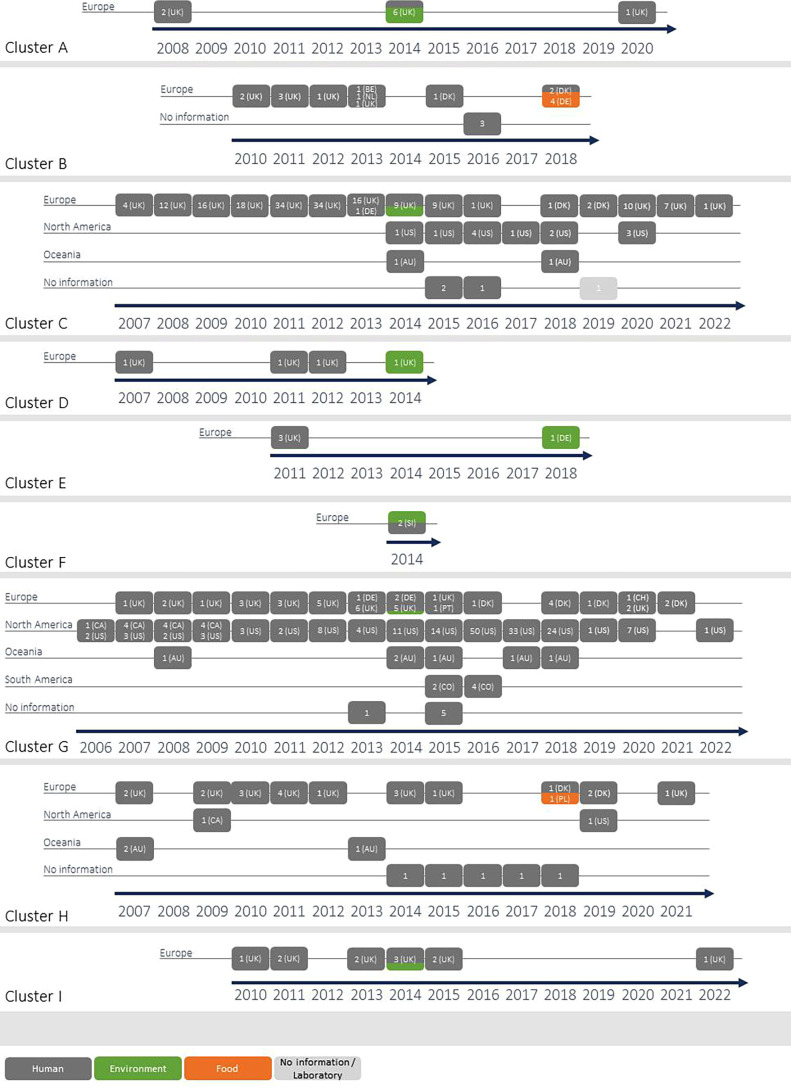
cgMLST ST8 clusters (threshold of three differing alleles) that contain *C. difficile* genomes derived from human and non-human samples. Only genomes with a given collection year are included in the figure. AU, Australia; BE, Belgium; CA, Canada; DE, Germany; DK, Denmark; NL, The Netherlands; PL, Poland; PT, Portugal; SI, Slovenia; UK, United Kingdom, US, United States of America.

With cgMLST, nine ST8/RT002 clusters (threshold three differing alleles) were detected that included genomes of human and non-human samples (Fig. 5). Furthermore, we investigated the antimicrobial resistance profiles of the genomes that were included in the following clusters.

Cluster A contained nine genomes from the UK from human (*n*=5) and wastewater (*n*=4) samples. According to cgMLST, the genomes from the wastewater samples showed no allelic differences.

Cluster B comprised 27 genomes in total, including four genomes from chicken meat products from Germany, 16 from human samples from Belgium, Denmark, the Netherlands, the UK and of unknown origin. Eight genomes (including one human sample) are not included in Fig. 5 as no year of isolation was given. Eleven genomes with no allelic differences according to cgMLST, including chicken wing samples from Germany, human samples from the UK and samples of unknown origin were detected; eight of them are shown in Fig. 5.

Cluster C contained 237 genomes, including human (*n*=208) and wastewater (*n*=5) samples, and samples of unknown origin (*n*=24). They originated from the UK (*n*=200), Denmark (*n*=3), Germany (*n*=1), the USA (*n*=12) and Australia (*n*=2), and were isolated between 2007 and 2022. The gene *erm*(B) was detected in one genome (human sample, UK, 2013). In five genomes, amino acid substitutions were detected in GyrA, with T82I in two genomes (one originated from the UK, no source mentioned) and D71V in three genomes (human sample, UK, 2011).

ST8 genomes from one wastewater and three human samples originating from the UK were assigned to cluster D. The genomes from human samples were detected in 2007, 2011 and 2012, and the one originating from the wastewater sample was detected in 2014. Three genomes (one wastewater and two human samples) showed an identical cgMLST profile.

Cluster E was composed of seven genomes. Three of them were detected in human samples in 2011 in the UK and one was detected in a chicken manure sample in 2018 in Germany. For three genomes from Germany, neither a year nor information on sampling were given. The genome from the chicken manure sample was identical to one of the genomes for which no information was given.

Two ST8 genomes from Slovenia (2014) belonged to cluster F. One derived from a human sample and the other one from a sample that was labelled as fish in Enterobase.

Cluster G contained 261 genomes, including human (*n*=249), one wastewater sample and 11 genomes of unknown origin. This cluster was widely spread, as the genomes originated from Europe, North and South America, and Oceania over a period of 17 years. The genome detected in the wastewater sample in 2014 was identical (according to cgMLST) to two genomes from human samples from the USA (2008) and Canada (2009). In 11 human genomes, additional antimicrobial resistances to the commonly detected ones (*lsa*, *bla*_CDD-1_, *blaR1*, *cdeA*, *vanG*, *vanRC*, *vanSC*, *vanTC* and *vanZA*) were found, including the substitution T82I in GyrA, together with *aadE* (one genome from the UK, 2010), the substitutions D71V in GyrA and D426N in GyrB (one genome from the UK, 2013), *tet*(M) (one genome from the USA, 2007), the substitution R447K in GyrB (two genomes from the USA, 2009 and 2014), the substitution D426N in GyrB (two genomes from the USA, 2013 and 2014), the substitution S550F in RpoB (two genomes from the USA, 2017 and 2018), the *dfrF* gene coding for a trimethoprim-resistant dihydrofolate reductase (one genome from the USA, 2018), and *aadE* (one genome from Denmark, 2018).

Cluster H was composed of 36 genomes and contained genomes from human samples (*n*=34), one genome that was isolated from a Polish potato sample, and one genome for which no information was available. The human samples originated from Australia (*n*=4), the UK (*n*=18), Denmark (*n*=3), Canada (*n*=1) and the USA (*n*=1); for seven genomes, no information was given.

Twelve genomes were assigned to cluster I, all originated from the UK. This cluster included genomes from 10 human samples, one wastewater sample and one sample of unknown origin. Ten genomes including the one from the wastewater sample were detected between 2010 and 2015, and one genome was detected seven years later in 2022. The wastewater sample genome and one of the human genomes from 2014 showed no allelic difference according to cgMLST.

## Discussion

### Overview of the *C. difficile* ST8/RT002 lineage

*C. difficile* ST8/RT002 is prevalent worldwide [9,12, 13, 16, 17, 63, 64] and can cause severe CDI [65,66]. This ST/RT has been shown to have a higher ability to cause clinically complicated infections compared to other RTs but has not provoked such severe disease as RT027 or RT078 [18]. As it is found in different animal species and environmental niches [67,71], a One Health approach is needed to understand the epidemiology and transmission routes of this clinically relevant RT lineage.

In this study, using a large collection of ST8/RT002, including *C. difficile* genomes collected from European countries (in the framework of the FED-AMR project) and genomes from the public database Enterobase from several geographical regions, we provided a detailed and comprehensive overview of the genomic features of this *C. difficile* lineage.

According to cgMLST and cgSNP analysis, we showed that RT002 genomes are closely related, as previously described [21,22, 64]. In our analysis, the genomes showed an average cgSNP distance of 71 SNPs and a median value of 67 SNPs. With cgMLST, the genomes revealed a difference of 44 alleles on average and a median cgMLST difference of 35. In a study of Baktash *et al.* [21], RT002 showed the lowest intra-RT allele difference for clade 1 with a mean value of 32 to 41 different cgMLST alleles depending on the core genome scheme used, and their SNP analysis revealed an average of 140 SNPs in the 12 investigated RT002 strains [21]. In a phylogenetic study by Eyre *et al.* [22], 44 European RT002 strains showed a median SNP value of 73 SNPs; furthermore, no within-country clustering was detected, but hospital-based clustering was reported [22]. Similar phylogenetic characteristics could be detected in the USA, where no obvious clustering by state was detectable [64]. However, in a study investigating RT002 genomes from different continents, a higher median pairwise SNP value of 258 was found [11]. Overall, previous studies reported core SNP data for RT002 ranging from 73 to 258, based on a set of genomes ranging from 12 to 79 genomes [11,21, 22, 64]; these results suggest that isolates of RT002 are genetically more closely related than other RT lineages and that related isolates can be detected independently from the geographical origin.

The results from the pangenome analysis indicate an open pangenome with an accessory genome that included 44.3 % of the pangenome. Compared to other *C. difficile* lineages, such as RT014 or ST11, with accessory genomes of 69.7 and 80.2 % [49,72], ST8/RT002 is more conserved. Nevertheless, it is important to be careful when comparing pangenome results from different studies involving other *C. difficile* lineages, as different software tools might be accountable for divergent results [49,72, 73]. However, even though the software panaroo delivers smaller pangenomes (*C. difficile* species pangenome with panaroo: 17, 470 genes; with Roary: 32, 802 genes), with a higher proportion of the core genome, the lineage ST8/RT002 still possessed an open genome as described for other *C. difficile* lineages and for the *C. difficile* species in general [72,75]. Additionally, differences in the gene cutoffs used to classify core and pangenomes, the number of genomes included, strain sources, diversity, and sampling strategy may influence the estimates of the pangenome.

### Virulence factors, antimicrobial resistance determinants and mobile genetic elements in *C. difficile* ST8/RT002

When we investigated the virulence factors of RT002, we noticed that parts of the binary toxin genes *cdtA* (coverage ranging from 33.62–37.86 %) and *cdtB* (coverage ranging from 31.28–59.60 %) were present but not those target sequences of common PCR primers [58]. Some RT lineages of clade 1 possess incomplete binary toxin genes as described previously [11]. The loss of toxin parts could indicate that the binary toxin does not play an essential role in general fitness of the RT lineage. In nearly all genomes, the virulence factors CD0873, CD2831, *fbpA*/*fbp68*, *groEL*, *zmp1*, *cwp84*, CD3246, *cbpA* and *cwpV* were present. These virulence factors play important roles – to a greater or lesser extent – in adhesion, colonization and biofilm formation [51,52, 54, 55, 57, 76,78]. *C. difficile* ST8/RT002 are clinically relevant [18,79], being commonly detected in CDI in cases of disease recurrence and in coinfections [63,80, 81].

In the analysed ST8/RT002 genomes, several AMR genes were detected that were present in all or most of the genomes. This includes *bla*_CDD_ genes and the multidrug efflux transporter *cdeA*, which are commonly found in *C. difficile* isolates [59,63]. Furthermore, the detected *van* gene cluster, *vanZA* and the *vga*(D) related gene were present in most of the ST8/RT002 isolates. The *van* gene cluster is not associated with a glycopeptide resistance phenotype [59]. Whether the *vga*(D) related gene confers resistance, and whether it is also commonly present in other RTs/STs, needs further investigation. Next to these genes, only few AMR determinants were found, mostly in strains from Asia. From the genomes that were included in the phylogenetic analysis, only 7.8 % (42/537) harboured antimicrobial resistance genes or mutations in *gyrA*, *gyrB* or *rpoB* leading to amino acid substitutions that are known to confer fluoroquinolone or rifampicin resistance [59]. This is in line with previous studies investigating antimicrobial resistance genes or antimicrobial susceptibility in RT002 compared to other RTs [11,20, 82]. This could be due to the generally low variation within this type and a particular stability. However, the AMR determinants detected in this study are able to confer resistance to different antimicrobial classes, including tetracyclines [*tet*(M) and *tet*(40)], MLS [*erm*(B), *cfr*(C) and *mef*(H)], aminoglycosides [*aadE* and *aac*(6′)-Ie/*aph*(2″)-Ia], fluoroquinolones (mutations in *gyrA* and *gyrB* genes), rifampicin (mutations in *rpoB*), and trimethoprim (*dfrF*). The presence of a broad range of AMR genes for different classes of antimicrobials and that genes of the same type were located on different putative mobile elements [e.g. *tet*(M) and *erm*(B)] are probably the result of horizontal gene transfers. This assumption is also supported by the finding showing that genomes of epidemiologically related clusters harboured different AMR determinants.

It was striking that 73 % of the Asian genomes harboured AMR determinants but only 4 and 7 % of the European and North American ones did. Moreover, a total of 18 of the 26 Asian genomes had substitutions in gyrase proteins that are known to confer fluoroquinolone resistance [59]. A high percentage of strains harbouring these mutations was also previously detected in RT002 genomes from PR China [83]. In addition, a relatively high percentage of moxifloxacin resistant isolates among ST8/RT002 isolates (ranging from 56–82 %) was also described for isolates from Japan [65]. The high percentage of fluoroquinolone-resistant isolates from Asia could be a result of antibiotic selection. Furthermore, a bias due to publishing only resistant isolates could be an explanation for these results.

With a customized database for MGEs (transposons, plasmids and prophages) that was created by literature search, we detected several MGEs. Tn*6189*, known to carry *erm*(B) [59], was the mostly detected transposon (18.6 %, 100/537). But it only carried *erm*(B) in eight cases. Genomes harbouring such a Tn*6189*-like element lacking *erm*(B) originated most often from Denmark (89 %, 82/92). Therefore, we assume that it is a locally evolved characteristic of these *C. difficile* ST8/RT002 isolates. This hypothesis is supported by the fact that from 2010 to 2017, few genomes harbouring this MGE were detected. In the following years, the number of genomes with this Tn*6189*-like element increased notably in Denmark. Several different plasmids were detected. As 87.7 % (471/537) of the genomes harboured the same five plasmids (pCDBI1, pAK2, pCDT4, pCD161-S and *C. difficile* strain FDAARGOS_267 plasmid 1), we had a closer look at the average nucleotide identity of these elements. The high similarity of the plasmids pCDT4, pCD161-S, pAK2 and *C. difficile* strain FDAARGOS_267 plasmid 1, as well as the high similarity of pAK1, pCD161-L and *C. difficile* strain FDAARGOS_267 plasmid 2, had already been detected in a previous study [84]. As plasmids with the same replication mechanism are normally unable to coexist in the same cell due to so called ‘incompatibility’ [85], it is possible that the corresponding region in the genomes was assigned to different, but highly similar, plasmids. A similar observation was made for the prophages phiCDHM11, phiCDHM13 and phiCDHM14. All genomes that contained phiCDHM14 harboured either phiCDHM11 or phiCDHM13 as well. These prophages belong to the same genus (phiMMP04likevirus) and are highly related [86]. Therefore, it is possible that the corresponding regions in the genomes were assigned to two different members of this prophage genus. MGEs in *C. difficile*, which comprise approximately 11 % of the genome [87], can transfer AMR and regulate toxin production [88]. Special attention and further research are needed to understand the influence of MGEs on the pathogenicity of *C. difficile* and its different lineages.

### Clusters of the ST8/RT002 lineage with One Health relevance

To investigate the zoonotic potential of ST8/RT002, we searched for genome clusters with genomes isolated from human and non-human samples. In a first step, we analysed the FED-AMR strain collection. Applying cgSNP and a threshold of two SNPs, we detected one cluster that included genomes of *C. difficile* isolates from human and chicken meat product samples. The isolates from the human samples were detected in Denmark and France from 2015 to 2017 and the isolates from the chicken meat products were detected in Germany in 2018. Four of these genomes (two human and two chicken product isolates) were even identical according to cgSNP (0 SNPs difference). Our results indicate that ST8/RT002 is a stable lineage with low variability. Clonality in terms of cgSNP differences therefore does not necessarily indicate a direct transmission. Although a direct zoonotic transmission cannot be proven, this cluster shows the zoonotic potential of ST8/RT002. Furthermore, we had a closer look at the MGEs in the genomes belonging to this cluster. Albeit identical according to cgSNP, different MGE profiles were detected in these genomes. Whether the assessment of MGEs in *C. difficile* can help to trace back outbreaks, especially in lineages with comparatively low allelic distances in the core genome, as is the case for ST8/RT002, needs further investigation.

Then, we investigated all available ST8/RT002 genomes (Enterobase and FED-AMR) for cross-species clusters through cgMLST analysis. Overall, nine clusters could be detected. In five of these clusters, isolates from wastewater samples (all from the UK) clustered with human samples (in three clusters, the human samples derived from the UK; in two clusters, they originated from different countries). In the other clusters, isolates from a potato sample, from chicken meat products, from chicken manure or from fish were present together with isolates from human samples. Although non-human genomes represented just 3.8 % of our ST8/RT002 collection, we were able to detect clusters that comprised 29 % (19/66) of these non-human samples. Three of these clusters (clusters C, G and H) were supra-continental. Cluster G even harboured identical strains according to cgMLST that were isolated over a period of seven years (2008–2014) in different continents, while cluster B spanned a period of nine years (2010–2018), also in different countries. From cluster I, two genomes were also investigated in the deeper analysis performed with the FED-AMR strains. The genomes from the wastewater and human sample revealed identical MGE profiles, indicating a very close genetic relationship. This shows the widespread occurrence and the possible ability of ST8 isolates to survive over a long time, putatively in humans as well as in the environment. Furthermore, several cases of re- and co-infection have been described in humans [63,66, 81]. The importance of this ability to persist in different ecological niches for the widespread distribution and a high prevalence of *C. difficile* has already been discussed by Janezic *et al.* [71]. The efficacy and characteristics of ST8/RT002 to form spores was investigated in some studies. ST8/RT002 showed a higher sporulation frequency compared to other ribotypes (with the exception of the hypervirulent RT027) and a better germination without taurocholate than other ribotypes, including RT027 [18,20]. These characteristics may play an important role in the epidemiology of ST8/RT002.

Even though ST8/RT002 is not classified as hypervirulent, and nor does it attract attention due to alarming antimicrobial resistance, it is a highly prevalent and clinically relevant RT lineage that is able to survive in several ecological niches. The generally low variation within this type, as well as the presence of clonal isolates in different compartments, time windows and regions, suggests, firstly, that this type has a particular genomic stability and, secondly, that there may even be a universal environmental source linking clonal isolates. Moreover, this stability does not seem to hinder the acquisition of AMR genes. Monitoring of this RT may reduce contamination and finally human and animal disease.

This analysis does not only present a comprehensive overview of ST8/RT002 genomes, but also highlights the importance of investigating non-human samples for a better understanding of epidemiological characteristics, i.e. contamination routes. Furthermore, it has been shown that ST8/RT002 is prevalent worldwide. Most of the investigated and available genomes originated from Europe and North America. Only few RT002 isolates originated from Oceania, Asia, South America and Africa [12,17, 63, 89], demonstrating the need for more widespread investigation in middle- and lower-income countries and analysis of isolates from these regions with next-generation sequencing. The intensive acquisition and study of non-human samples for a comprehensive picture of this pathogen in the context of One Health is mandatory.

**Impact Statement**

*Clostridioides difficile* is an important enteropathogen that can cause severe infections. *C. difficile* ribotype (RT)002, mainly associated with MLST sequence type (ST) 8, is one of the most commonly detected RTs in human samples, but is also detected in animals and in the environment. This is the first study that focuses on RT002/ST8 by making use of an exhaustive European isolate collection and all available ST8 genomes from the public database Enterobase. We were able to identify clusters that included strains of human and non-human origin and could prove that RT002/ST8 is a stable but adaptable lineage of *C. difficile*. With this study, we provide a comprehensive genomic overview of this globally prevalent RT lineage and show the importance of a One Health approach to combat human infections with this notorious antimicrobial-resistant pathogen.

## supplementary material

10.1099/mgen.0.001270 Uncited Fig. S1.

10.1099/mgen.0.001270 Uncited Table S1.

## Abbreviations

AMR: antimicrobial resistance
CDI: clostridioides difficile infection
cgMLST: core genome multilocus sequence typing
cgSNP: core genome single nucleotide polymorphism
COG: cluster of orthologous groups
HMM: Hidden Markov Models
MGE: mobile genetic element
MLST: multilocus sequence typing
RT: ribotype
ST: sequence type
WGS: whole genome sequencing
