# Supplementary material for: Genomic study of European Clostridioides difficile ribotype 002/sequence type 8
Source: Microb Genom. 2024 Jul 25;10(7):001270. doi: 10.1099/mgen.0.001270 (PMC11316560; doi:10.1099/mgen.0.001270)
Supplement: Uncited Fig. S1. [file mgen-10-01270-s001.pdf]

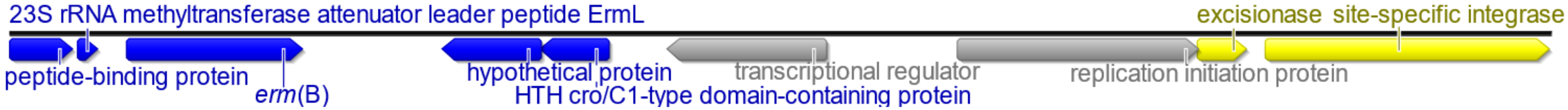

Figure S1: Schematic presentation of the mobile genetic element (MGE) containing erm(B) in CLO_EA0075AA. Elements of Tn*6189* are shown in blue, elements of Tn*6218* in yellow. The site-specific integrase and the excisionase showed identities of 95.3 % and 94.2 % to the respective genes in Tn*6218*. This figure was created with Geneious version 2021.0 created by Biomatters (available from https://www.geneious.com).

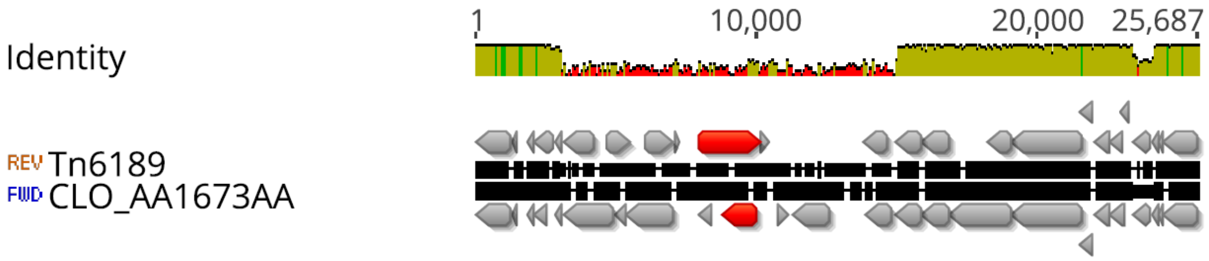

Figure S2: Schematic presentation of the mobile genetic element (MGE) containing cfr(C) in CLO_AA1673AA. Grey arrows indicate coding regions. The antimicrobial resistance genes *cfr*(C) (CLO_AA1673AA) and *erm*(B) (Tn*6189*) are shown in red. The black bars display whether there are nucleotides at this position in the respective sequence. Green in the identity graph means complete identity in this area, yellow means less than complete identity, and red stands for very low identity. This figure was created with Geneious version 2021.0 created by Biomatters (available from https://www.geneious.com).

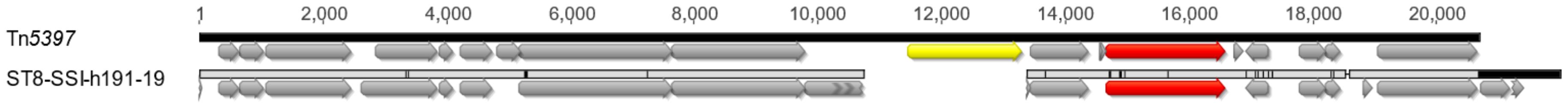

Figure S3: Schematic presentation of the mobile genetic element (MGE) containing tet(M) in ST8-SSI-h191-19. Grey arrows indicate coding regions. The antimicrobial resistance genes *tet*(M) are shown in red. The group II intron (Tn*5397*) is shown in yellow. Nucleotide disagreements of the MGE in ST8-SSI-h191-19 in comparison to Tn*5397* are displayed in black in the grey bar. For a better comparison of the two MGEs, the MGE of ST8-SSI-h191-19 was cut into two parts. This figure was created with Geneious version 2021.0 created by Biomatters (available from https://www.geneious.com).

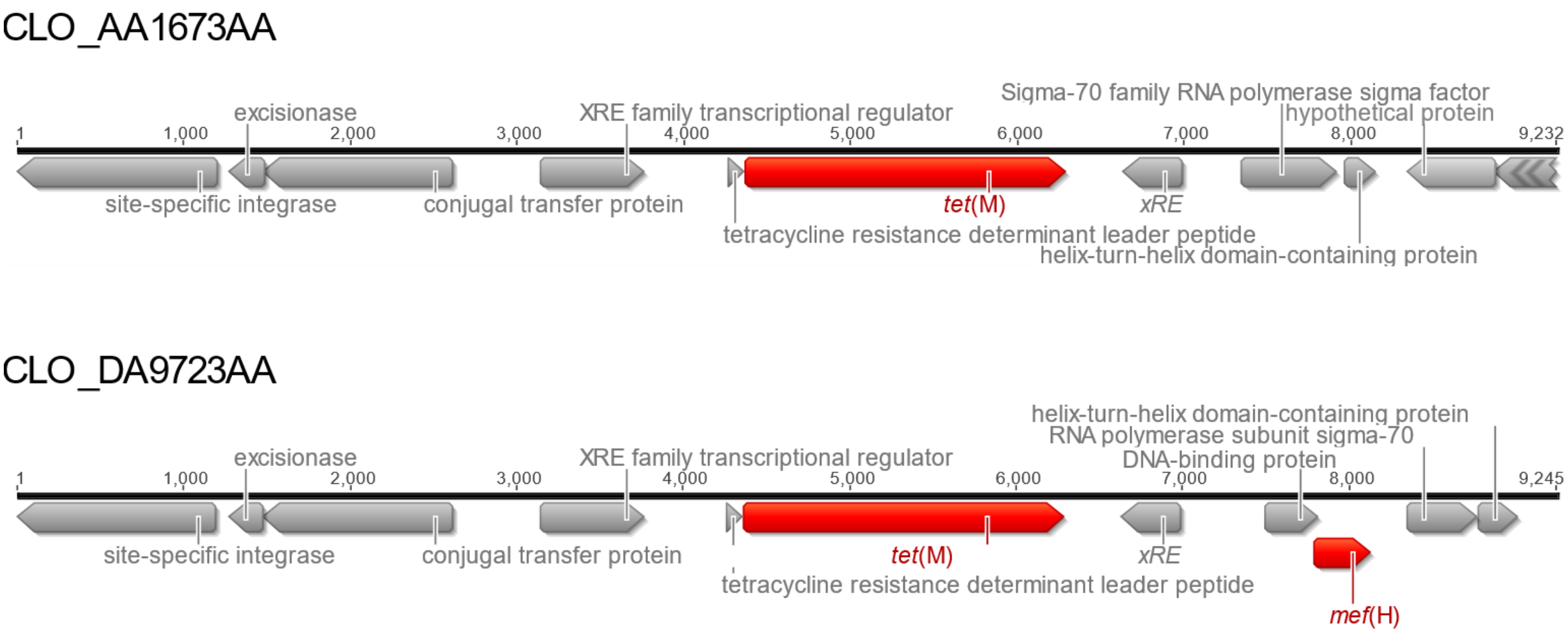

Figure S4: Schematic presentation of the mobile genetic elements (MGEs) containing tet(M) in CLO_AA1673AA and CLO_DA9723AA. Grey arrows indicate coding regions. The antimicrobial resistance genes *tet*(M) and *mef*(H) are shown in red. This figure was created with Geneious version 2021.0 created by Biomatters (available from https://www.geneious.com).

A

B

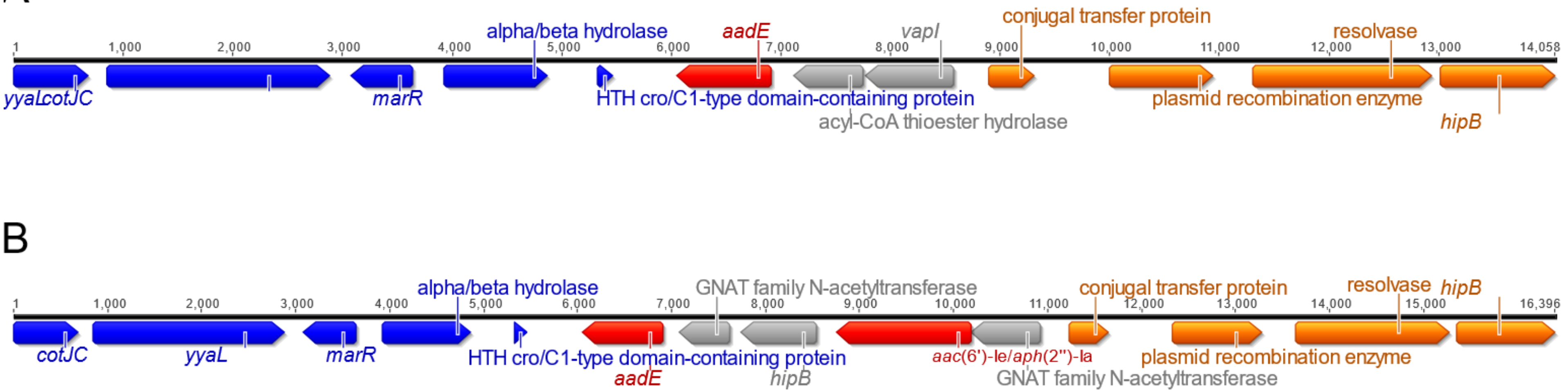

Figure S5: Schematic presentation of the mobile genetic elements (MGEs) containing aadE and aac(6')-Ie/aph(2")-Ia. Arrows indicate coding regions. The areas highlighted in blue and orange were identical in all six elements except for a single SNP in the plasmid recombination genes of the MGEs containing *aadE* and *aac*(6')-Ie/*aph*(2")-Ia compared to the MGEs that only harboured *aadE*. The antimicrobial resistance genes *aadE* and *aac*(6')-Ie/*aph*(2")-Ia are shown in red. A: MGE containing *aadE* in CLO_DA7520AA, DSM29687 and ST8-SSI-h020-18. B: MGE containing *aadE* and *aac*(6')-Ie/*aph*(2")-Ia in CLO_DA9523AA, CLO_DA9620AA and CLO_DA9627AA. This figure was created with Geneious version 2021.0 created by Biomatters (available from https://www.geneious.com).

Figure S6A

Average Nucleotide Identity of Transposons identified in *Clostridioides difficile*

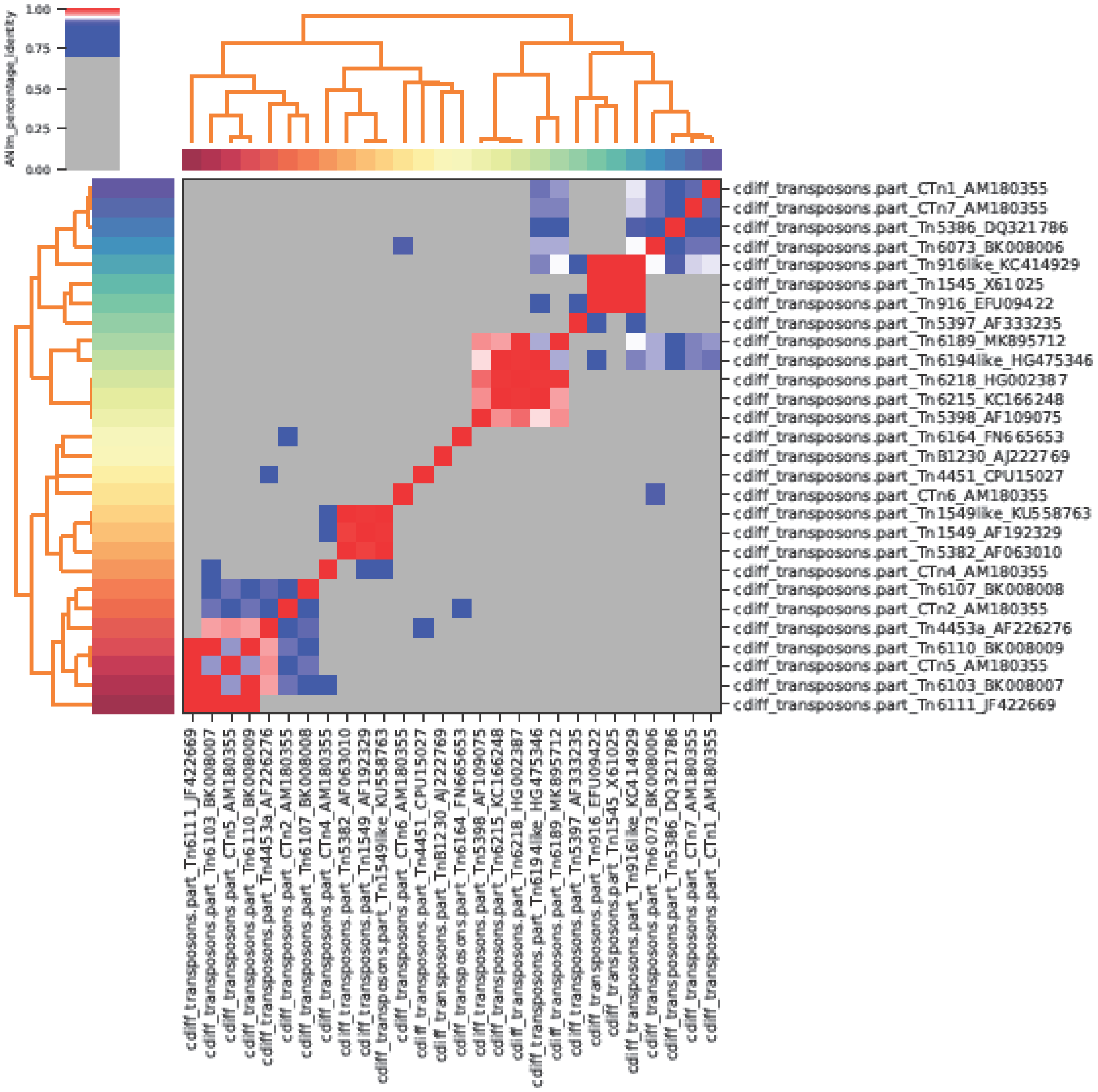

Figure S6B

Average Nucleotide Identity of Plasmids identified in *Clostridioides difficile*

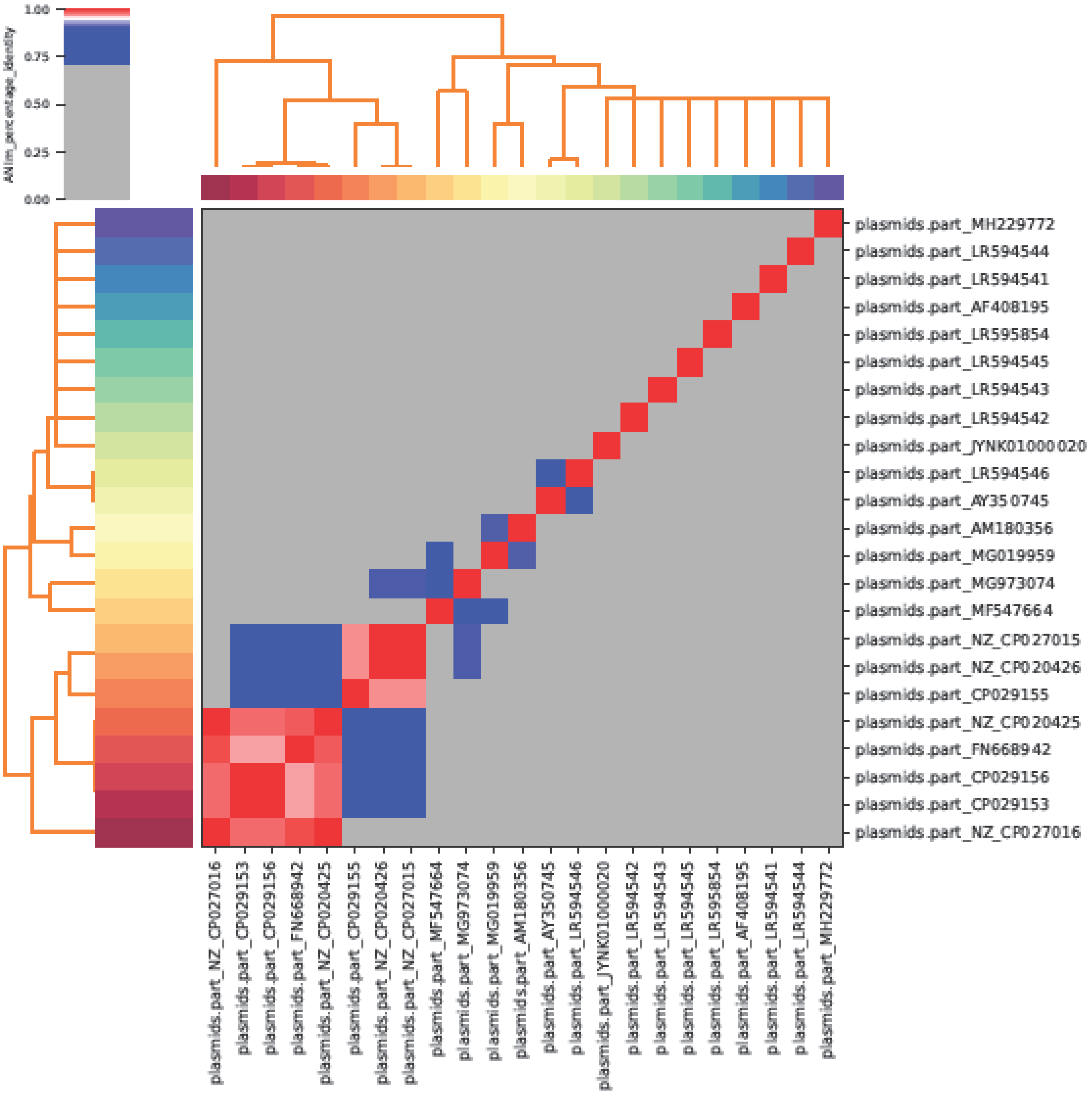

Figure S6C

Average Nucleotide Identity of Phages identified in *Clostridioides difficile*

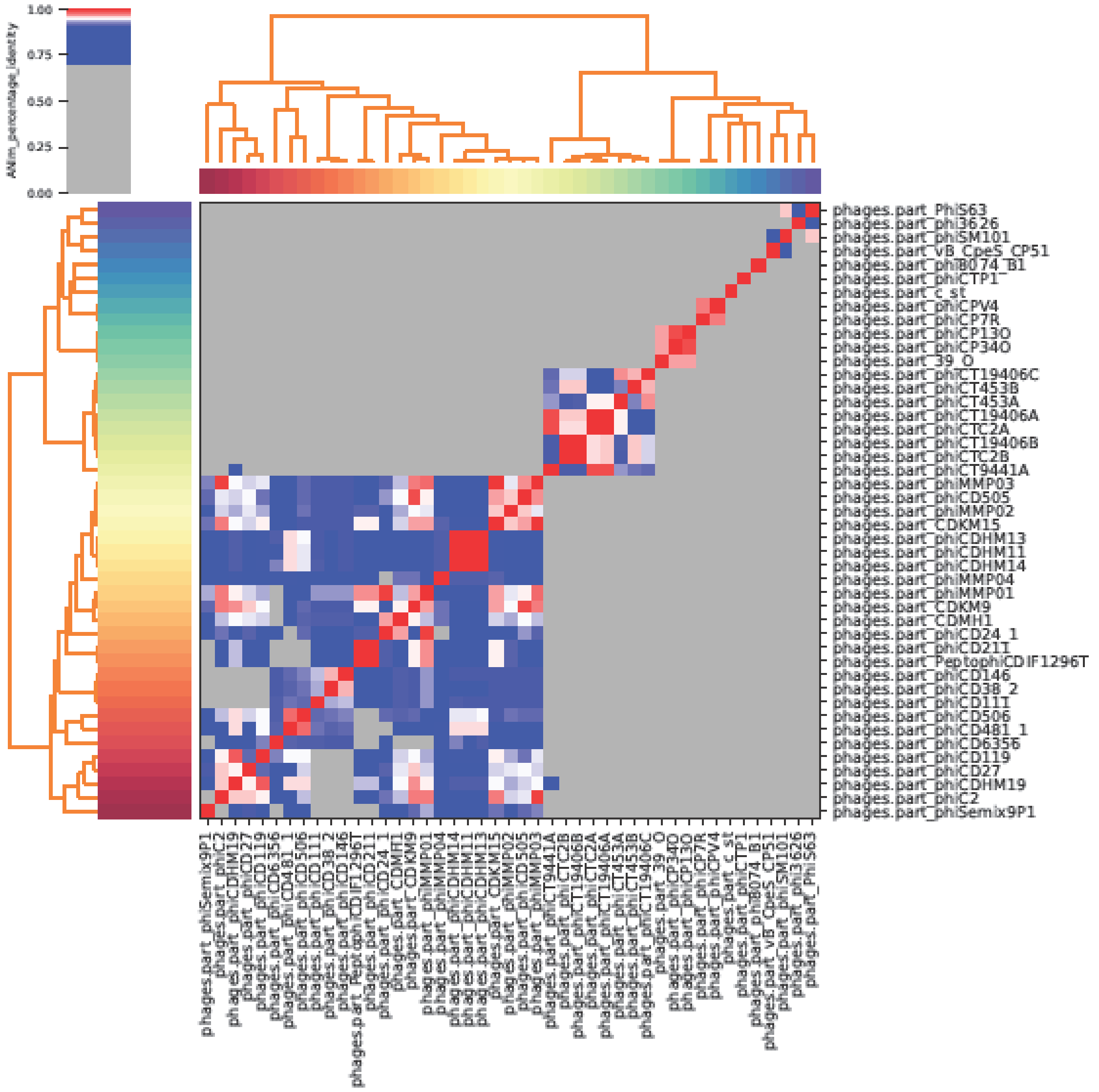

Figure S7

Spatio-temporal abundance of ST8 / RT002 in Enterobase given in percentage and absolute numbers per continent and year

	1900-2005	2006	2007	2008	2009	2010	2011	2012	2013	2014	2015	2016	2017	2018	2019	2020	2021	2022	No information	Total
Africa	-	-	-	-	-	-	-	0% (0/4)	-	-	-	-	-	-	-	-	-	-	-	0% (0/4)
Asia	0% (0/34)	0% (0/2)	0% (0/3)	0% (0/8)	4% (1/25)	0% (0/20)	0% (0/29)	0% (0/15)	13% (2/16)	8% (7/83)	11% (24/224)	11% (10/89)	2% (4/206)	0% (0/23)	-	-	-	-	0% (0/94)	6% (48/871)
Europe	1% (1/111)	1% (2/154)	3% (19/601)	4% (28/636)	5% (38/698)	7% (54/789)	8% (114/1362)	7% (90/1324)	8% (71/915)	6% (68/1218)	4% (28/658)	1% (3/223)	3% (3/104)	5% (5/106)	4% (4/109)	10% (27/264)	13% (26/207)	7% (7/96)	5% (136/2909)	6% (724/12484)
North America	0% (0/122)	6% (4/68)	6% (15/241)	5% (11/220)	9% (14/159)	2% (6/253)	2% (7/327)	5% (19/352)	5% (20/393)	7% (26/380)	7% (25/383)	8% (114/1373)	7% (92/1254)	8% (45/532)	3% (8/280)	5% (19/400)	0% (0/11)	50% (1/2)	1% (2/178)	6% (428/6928)
Oceania	0% (0/14)	0% (0/18)	15% (3/20)	19% (3/16)	0% (0/2)	0% (0/8)	0% (0/15)	0% (0/79)	2% (1/43)	20% (8/41)	6% (2/36)	24% (9/38)	9% (3/34)	12% (4/33)	0% (0/34)	-	-	-	1% (1/118)	6% (34/549)
South America	0% (0/4)	-	-	-	0% (0/9)	-	0% (0/6)	-	0% (0/2)	-	17% (2/12)	22% (9/41)	0% (0/3)	5% (1/20)	0% (0/16)	-	-	-	-	11% (12/113)
No information	5% (1/22)	0% (0/2)	0% (0/8)	0% (0/5)	0% (0/18)	3% (1/31)	0% (0/5)	0% (0/16)	6% (3/54)	20% (17/84)	7% (16/234)	13% (17/135)	13% (13/98)	33% (8/24)	5% (2/42)	0% (0/5)	0% (0/3)	-	5% (113/2278)	6% (191/3064)
Total	1% (2/307)	2% (6/244)	4% (37/873)	5% (42/885)	6% (53/911)	6% (61/1101)	7% (121/1744)	6% (109/1790)	7% (97/1423)	7% (126/1806)	6% (97/1547)	9% (162/1899)	7% (115/1699)	9% (63/738)	3% (14/481)	7% (46/669)	12% (26/221)	8% (8/98)	5% (252/5577)	6% (1437/24013)

Colour coding: The results in this table are coloured in blue / purple tones according to the abundance of ST8 / RT002 in Enterobase per continent and year:

0% 1% 30%
